# The phenotypic and genotypic antimicrobial resistance patterns of *Salmonella* isolated from chickens and meat at poultry slaughterhouses in Japan and Thailand

**DOI:** 10.14202/vetworld.2023.1527-1533

**Published:** 2023-07-24

**Authors:** Pattarakitti Noenchat, Kochakorn Direksin, Pairat Sornplang

**Affiliations:** 1 Sakon Nakhon Provincial Livestock Office, Department of Livestock Development, Ministry of Agriculture and Cooperatives, Sakon Nakhon 47000, Thailand; 2 Division of Veterinary Public Health, Faculty of Veterinary Medicine, Khon Kaen University, Khon Kaen 40002, Thailand; 3 Division of Livestock Medicine, Faculty of Veterinary Medicine, Khon Kaen University, Khon Kaen 40002, Thailand

**Keywords:** antimicrobial resistance, broiler chickens, meat, *Salmonella*, slaughterhouse

## Abstract

**Background and Aim:**

Poultry meat is a popular food consumed globally. However, poultry farming is a source of *Salmonella* contamination which causes human salmonellosis. This study aimed to estimate the prevalence and antimicrobial resistance (AMR) of *Salmonella* among chickens and meat at poultry slaughterhouses in province study areas in Thailand and Japan.

**Materials and Methods:**

Chicken meat and feces samples were collected in Thailand and Japan. In Nakhon Ratchasima Province, Thailand, 558 samples were obtained from slaughterhouses from January 2021 to March 2022. In Gifu Prefecture, Japan, 140 samples (70 each of intestinal contents and meat) were purchased from slaughterhouses from June to October 2022. For *Salmonella* detection, the samples were cultivated according to the International Organization for Standardization 6579:2002/AMD 1:2007 method and confirmed using polymerase chain reaction (PCR) and agglutination tests for serotyping. Isolated *Salmonella* were tested for AMR to nine antibiotics using the disk diffusion method. Selected phenotypic multidrug-resistant (MDR) isolates were evaluated for AMR genes (AMRGs) using PCR.

**Results:**

*Salmonella* prevalence from chickens and meat at slaughterhouses in Thailand and Japan was 41.2% and 40.7%, respectively. All the *Salmonella* isolates in Japan were serotyped as Schwarzengrund, and no *Salmonella* isolates were resistant to the nine antibiotics tested. In contrast, most of the Thai *Salmonella* isolates from chicken cloacal swabs and meat were resistant to doxycycline (78.3%) and colistin (63.5%). The prevalence of MDR *Salmonella* (MDRS) in chickens and meat from Thailand and Japan was 29.1% (67/230) and 0% (0/57), respectively. However, the most frequent AMRGs found in MDRS in Thailand were extended-spectrum beta-lactamase-Temoneira (ESBL-TEM) (45.1%). All isolated *Salmonella* from Japan revealed a class 1 integron gene (*Int-1*).

**Conclusion:**

Phenotypic MDRS isolates from Thailand showed the greatest correlation to AMRG and ESBL-TEM. Although there were no phenotypic AMR *Salmonella* isolates found in Japan, they can be found associated with *Int-1*, which may carry other AMRGs within the gene cassettes.

## Introduction

Poultry farming is conducted widely in Thailand at both small farm and industrial levels. Poultry meat is popularly consumed and generally distributed throughout the Thai market. In addition, in 2022, Thailand ranked fourth among the world chicken exporters of processed chicken meat. Notably, Nakhon Ratchasima Province is among the three provinces with the highest broiler production in Thailand. In addition, the number of Japanese who consumed chicken meat dramatically increased from 2015 to 2021. Furthermore, Japan is the primary importer of chicken meat from Thailand. However, poultry and poultry products have been proposed as a primary source of bacterial contamination originating from poultry farms or during slaughtering, which causes human foodborne illnesses such as salmonellosis through meat consumption [1, 2]. In addition, antibiotic-resistant bacteria originating from bacterial infections could remain in the human body through food consumption, posing a global public health concern because of treatment difficulties.

Recently, researchers have described the prevalence and antimicrobial resistance (AMR) of *Salmonella* in broiler chickens in Thailand [3] and Japan [4, 5]. However, little is known about *Salmonella* prevalence in broiler chickens and their potential AMR at domestic (or local) poultry slaughterhouses based on good manufacturing practice (GMP) usage for hygiene systems in these two countries. Several studies in Thailand and Japan have detected AMR *Salmonella* at a phenotypic level, but its correlation to a genotypic level is limited [6–8]. In addition, most AMR genes (AMRGs) found in *Salmonella*, including extended-spectrum beta-lactamase (ESBL), colistin (CT), and integrons, have been recently reported as the prevalence varies depending on sample types, regional studies, and serovar finding [9].

Therefore, this study aimed to identify *Salmonella* isolates from broiler chickens and their products from poultry slaughterhouses in Nakhon Ratchasima Province, Thailand, and Gifu Prefecture, Japan. This study also estimated the prevalence of AMR, multidrug-resistance (MDR), and AMRGs in positive *Salmonella* isolates from both countries.

## Materials and Methods

### Ethical approval

The sampling of preslaughtered broiler chickens from Thailand in the present study was approved under the permissions and guidelines of the Institutional Animal Care and Use Committee of Khon Kaen University (KKU), Thailand (permission record no. IACUC-KKU-79/64). In Japan, samples (intestinal contents/chicken meat) were purchased from slaughtered broiler chickens at a local poultry slaughterhouse. Therefore, ethical approval for animal use was not needed.

### Study period and location

The study was conducted from January 2021 to October 2022. The samples were obtained from local poultry slaughterhouses in Thailand and Japan. The samples were processed at Khon Kaen University for Thai AMR Salmonella isolates, and at Gifu University for Japanese AMR Salmonella isolates.

### Sampling

Samples were obtained using a cross-sectional design. We collected 279 cloacal swab (preslaughter) samples of chickens and 279 chicken breast meat (post-slaughter) samples, to make a total of 558 samples. The samples were collected from poultry slaughterhouses in Nakhon Ratchasima Province, Thailand, from January 2021 to March 2022. All the farms and slaughterhouses were certified to raise and slaughter per GMP by the Department of Livestock Development, Thailand. Based on the 41.6% *Salmonella* prevalence found in chicken meat in a previous study by Shimojima *et al*. [10] in Japan, the calculated sample size could be ≥389 samples collected from Japan. Due to slaughterhouse regulatory restrictions, we were only allowed to enter and purchase a total of 140 samples (70 chicken intestinal content samples and 70 breast meat samples). The samples were purchased from five broiler flocks at pre- and post-slaughter periods from one poultry slaughterhouse in Gifu Prefecture from June 2022 to October 2022. All the farms and slaughterhouses have also been granted GMP certificates by the Japanese government.

Fresh fecal samples from chicken cloacal swabs from Thailand and chicken intestinal contents from Japan (because live chickens were not allowed to approach) were randomly and aseptically collected from poultry slaughterhouses. Chicken breast meat samples were obtained from the poultry slaughterhouse. Each sample was placed in a sterile sample bag, maintained at 4°C, and delivered to the bacterial laboratory within 24 h for further bacterial isolation.

### Sample preparation and *Salmonella* isolation

*Salmonella* isolation in a pre-enrichment step was performed by mixing approximately 1 g of chicken feces from intestinal contents or cloacal swab samples in 10 mL of sterile buffered peptone water (BPW; Oxoid, Hampshire, UK) and then incubated at 37°C for 16–24 h. A total of 25 g of chicken breast meat was mixed in 225 mL of BPW (Oxoid, Basingstoke, UK) and then incubated at 37°C for 16 h–24 h.

*Salmonella* detection was further conducted using the ISO 6579: 2002/AMD 1:2007 method. Briefly, 100 μL aliquots of the pre-enrichment samples were inoculated with three pipette drops onto Modified Semi-solid Rappaport Vassiliadis medium (MSRV; Oxoid) and then incubated at 42°C for 18 h–24 h. After enrichment**,** one loop of MSRV was streaked onto xylose lysine deoxycholate agar (XLD; Oxoid, USA) and Hektoen enteric agar (HE; Oxoid) and then incubated at 37°C for 18 h–24 h. Up to five typical *Salmonella* colonies from XLD and HE (black‒green colonies) were selected and streaked on nutrient agar per plate (NA; Oxoid) and incubated at 37°C for 16 h–24 h to biochemically confirm *Salmonella*. Briefly, selected colonies from NA were transferred onto triple sugar iron agar (TSI; Oxoid) and motility indole lysine medium (MIL; Oxoid). These colonies were incubated at 37°C for 18–24 h. Next, positive colonies from TSI and MIL tests were placed in sterile cryovial tubes with stock medium (600 µL BPW and 200 µL 20% glycerol), stored at −80°C, and further tested.

### Molecular detection of *Salmonella* and serotyping

Deoxyribonucleic acid (DNA) from positive *Salmonella* cultivation from Japan was extracted as described by Gebeyehu *et al*. [11]. The primers used for amplification were Salm3 (forward): 5’GCT GCG CGC GAA CGG CGA AG 3’ and Salm4 (reverse): 5’TCC CGG CAG AGT TCC CATT 3’. The polymerase chain reaction (PCR) was conducted with an initial denaturation at 95°C for 5 min, followed by 35 cycles of denaturation at 95°C for 1 min, annealing at 65°C for 1 min, and extension at 72°C for 1 min with a final additional extension for 7 min at 72°C. This was followed by storage at 4°C. A 2% agarose gel was prepared and electrophoresed. Our next step was to view it through ultraviolet transillumination and analyze it based on a prior study by Gebeyehu *et al*. [11]. One to two confirmative *Salmonella* colonies selected from both Thai and Japanese isolates from each sample were grown on NA to allow for *Salmonella* serotyping according to the White-Kauffmann-Le Minor method using slide agglutination with O and H antigen-specific sera (Denka Seiken Co., Ltd., Tokyo, Japan). Nuclease-free water and a *Salmonella* reference strain (obtained from the Graduate School of Veterinary Science, Gifu University, Japan) were used as negative and positive controls for the PCR procedure, respectively.

### Antimicrobial susceptibility test

The *Salmonella* positive samples were evaluated for antimicrobial susceptibility using the Kirby-Bauer disk diffusion method [12]. Next, the antimicrobial susceptibility of these isolates was interpreted using Clinical and Laboratory Standards Institute guidelines [13]. Subsequently, nine antimicrobial agents from five different antibiotic classes commonly used for treating diseases in humans and animals were investigated: beta-lactam (amoxicillin 10 μg, ampicillin 10 μg, cefotaxime 30 μg, cefepime 30 μg, and cefoxitin 30 μg), chloramphenicol (C, 30 μg), peptide (CT, 10 μg), tetracycline (doxycycline [DO], 30 μg), and sulfonamide (sulfamethoxazole/trimethoprim [19:1] 25 μg). All antimicrobial disks were obtained from Eiken (Eiken Chemical Co., Ltd., Tokyo, Japan).

### Prepared bacterial inoculum

A selected *Salmonella* colony was suspended in 2 mL of 0.85% (w/v) normal saline. Next, the inoculum was adjusted to a turbidity equivalent of a 0.5 McFarland standard (1.5 × 10^8^ colony-forming unit/mL). Then, the inoculum was dipped using a sterile cotton swab and applied onto a Mueller-Hinton agar (Becton Dickinson, Franklin Lakes, NJ, USA) plate. This was followed by incubation under aerobic conditions at 37°C for 48 h. *Escherichia coli* ATCC25922 was used as the quality control.

### Detection of AMRGs

Phenotypically selected MDR *Salmonella* (MDRS) isolates from each poultry slaughterhouse were further evaluated for the presence of three AMRGs: Extended-spectrum beta-lactamase-Temoneira (ESBL-TEM), Class 1 integron (*Int-1*), and CT (*mrc-1*). These genes are commonly found in pathogenic bacteria. Deoxyribonucleic acid was extracted and purified from the overnight culture of the positive phenotypic MDRS isolates using a FastGene Gel/PCR extraction kit (Nippon Genetics Europe, Düren, Germany) according to the manufacturer’s instructions. Polymerase chain reaction was conducted in 25 mL of the reaction mixture containing bacterial cells as the source of the DNA template, *Taq* DNA polymerase (Merck KGaA, Darmstadt, Germany), PCR-grade water (Thermo Fisher Scientific [Thailand] Co., Ltd., Bangkok, Thailand), and specific primer pairs (Thermo Fisher Scientific [Thailand] Co., Ltd.,). Thermocycling followed the PCR conditions of the gene primers used and are listed in Table-1 [14, 15]. A 2% of agarose gel was prepared and electrophoresed using 0.5 M Tris-borate-ethylenediamine tetraacetic acid buffer. Electrophoresis was performed at 76 V (constant voltage) for approximately 30 min. The gels were stained using an ethidium bromide solution (5 mg/mL) for 20 min, washed with deionized water, and viewed using ultraviolet transillumination.

**Table-1:** The three-gene specific primers used in this study.

Gene	Sequence (5’–3’) (F=Forward, R=reverse)	Annealing temperature (°C)	Amplicon size (bp)	Reference
ESBL-TEM	F-TTTCGTGTCGCCCTTATTCC	50	404	[14]
ESBL-TEM	R-ATCGTTGTCAGAAGTAAGTTGG	50	404	[14]
*Int-1*	F-GGGTCAAGGATCTGGATTTCG	62	483	[15]
*Int-1*	R-ACATGGGTGTAAATCATCGTC	62	483	[15]
*mcr-1*	F-AGTCCGTTTGTTCTTGTGGC	58	320	[15]
*mcr-1*	R-AGATCCTTGGTCTCGGCTTG	58	320	[15]

ESBL-TEM=Extended-spectrum beta-lactamase–Temoneira, *Int-1**=***Class 1 integron gene

### Statistical analysis

The MDR pattern is described as having resistance to antibiotics from a minimum of three antimicrobial classes. First, positive *Salmonella* isolates from chicken cloacal swabs or intestinal contents, chicken breast meat, and phenotypic/genotypic AMR profiles were reported in percentages. Then, the Chi-square test was used to analyze the correlation between the diverse prevalence of *Salmonella* isolates in chicken cloacal swabs or intestinal contents and chicken breast meat samples and their phenotypic/genotypic AMR profiles. Finally, statistical analyses were performed using statistical package for the social sciences (SPSS) for Windows (ver. 16.0; SPSS Inc., Chicago, IL, USA), and p < 0.05 was considered statistically significant.

## Results

### Prevalence of the *Salmonella*

Of the 558 samples investigated, 230 Thai chicken and chicken meat samples were *Salmonella* positive (41.2%), while *Salmonella* isolates were recovered from 40.7% (57/140) of the Japanese samples. The prevalence of Thai *Salmonella* in broiler chicken meat (48%, 134/279) was greater than that in broiler chicken cloacal swab samples (34.4%, 96/279). In contrast, in Japan, the *Salmonella* prevalence observed in broiler chicken meat (32.9%, 23/70) was less than that observed in broiler chicken intestinal content samples (48.6%, 34/70) (Table-2). In Thailand, the most common *Salmonella* positive serovars found in cloacal swab samples were Enteritidis (73.36%), Typhimurium (15.63%), and Mbandaka (10.42%). *Salmonella* serovars from breast meat samples included Corvallis, Enteritidis, Typhimurium, Mbandaka, and Braenderup at 44.78%, 20.15%, 14.93%, 11.19%, and 8.96%, respectively. All Japanese *Salmonella* isolates were serotyped as Schwarzengrund (Table-3).

**Table-2:** The prevalence of *Salmonella* isolated from chicken and chicken meat at local poultry slaughterhouses in Thailand and Japan.

Country	Sample	Positive-*Salmonella* isolates (%)
Thailand	CS^1^	96/279 (34.4)
	BM^2^	134/279 (48.0)
Total		230/558 (41.2)
Japan	IC^3^	34/70 (48.6)
	BM	23/70 (32.9)
Total		57/140 (40.7)

1 Cloacal swab,

2 Intestinal content,

3 Breast meat

**Table-3:** Serotyping of *Salmonella* positive samples.

Country	Sample	No. of *Salmonella* isolates	Serotypes (%)					

			Braenderup	Corvallis	Enteritidis	Mbandaka	Schwarzengrund	Typhimurium
Thailand	CS^1^	96	0 (0)	0 (0)	71 (73.96)	10 (10.42)	0 (0)	15 (15.63)
	BM^2^	134	12 (8.96)	60 (44.78)	27 (20.15)	15 (11.19)	0 (0)	20 (14.93)
Japan	IC^3^	34	0 (0)	0 (0)	0 (0)	0 (0)	34 (100)	0 (0)
	BM	23	0 (0)	0 (0)	0 (0)	0 (0)	23 (100)	0 (0)

1 Cloacal swab,

2 Intestinal content,

3 Breast meat

### Phenotypic and genotypic AMR profiles of the *Salmonella*

**Figure-1:**
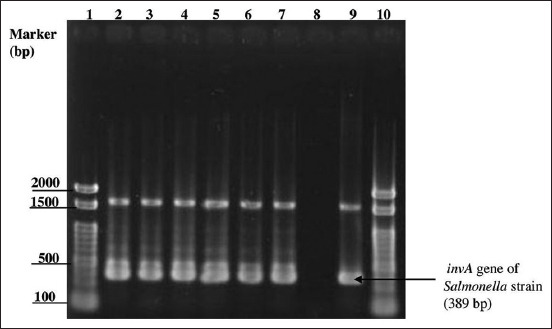
Multiplex polymerase chain reaction products with *invA* primers (389 bp), Lanes: 1 and 10, 100 bp ladder marker (Thermo Fisher Scientific, St. Leon-Rot, Germany); 2–7, field samples; 8, nuclease free water (negative control); 9, *Salmonella* Schwarzengrund obtained from Graduate School of Veterinary Science Gifu University, Japan (positive control).

**Figure-2:**
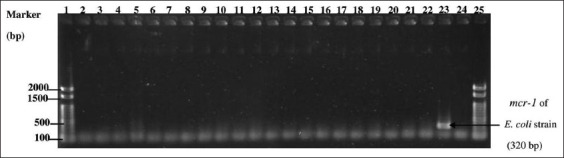
Multiplex polymerase chain reaction products with *mcr-1* primers (320 bp). Lanes: 1 and 25, 100 bp ladder marker (Thermo Fisher Scientific, St. Leon-Rot, Germany); 2–22, field samples; 23, *Escherichia coli* strain obtained from Graduates School of Veterinary Science, Gifu, University, Japan (positive control); 24, nuclease free water (negative control).

**Figure-3:**
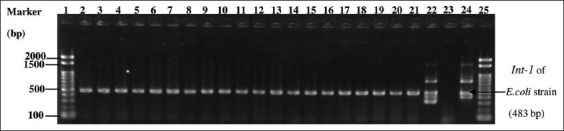
Multiplex polymerase chain reaction products with *Int-1* primers (483 bp). Lanes: 1 and 25, 100 bp ladder marker (Thermo Fisher Scientific, St. Leon-Rot, Germany); 2–21, field samples; 22 and 24, *Escherichia coli* strains obtained from Graduates School of Veterinary Science, Gifu, University, Japan (positive control); 23, nuclease free water (negative control).

Most *Salmonellae* isolated from Thai cloacal swabs and breast meat samples were phenotypically resistant to DO (84.37% and 73.88%, respectively) and CT (68.75% and 59.7%, respectively). In contrast, Japanese *Salmonella* isolates had no phenotypic resistance to any tested antibiotic. However, all these positive *Salmonellae* from chicken intestinal contents and breast meat had intermediate resistance to DO at 85.29% and 91.30%, respectively (Table-4). Of the 230 phenotypically resistant isolates from Thailand, 70 were MDRS. In addition, the MDRS isolates from the chicken cloacal swabs (33/70) were not observed to be significantly different from those found in chicken breast meat (34/70) (p = 0.135). They were also found to be resistant to 3–6 antimicrobial agents from 3 to 5 antimicrobial classes (Table-5). In contrast, no MDRS isolates were identified among the Japanese *Salmonella* isolates. Subsequently, in the Thai sampling, 50 of the 70 MDRS isolates from both cloacal swabs and breast meat samples from all 31 slaughterhouses were selected and tested for three AMRGs. The selected phenotypic MDRS isolates most commonly contained ESBL-TEM (43.48%, 46.43%), and *mrc*-*1 (*14.28%, 8.69%). In Japan, all *Salmonella* isolates harbored *Int-1* genes (Table-6). PCR amplicons on agarose gels of Japanese *Salmonella* positive isolates, *mrc*-*1*, and *Int-1* genes are shown in Figures-1–3, respectively.

**Table-4:** Antimicrobial resistance and intermediate susceptibility of *Salmonella* isolates from chickens and meat at local poultry slaughterhouses in Thailand and Japan.

Country	Sample	No. of *Salmonella* isolates	Antimicrobial agents								

			AML	AMP	C	CT	CTX	DO	FEP	FOX	SXT
Thailand	CS^1^	96	25R	2I; 26R	4I; 12R	1I; 66R	2R	1I; 81R	5I; 1R	3I; 1R	28R
	BM^2^	134	18R	7I; 17R	10I; 14R	8I; 80R	5I	1I; 99R	3I; 2R	3I; 1R	27R
Total RS^3^ (%)			18.69	18.69	11.3	63.48	0.87	78.26	1.3	0.87	23.91
Japan	IC^4^	34	0	0	0	0	0	29I	0	0	0
	BM	23	0	0	0	0	0	21I	0	0	0
Total RS (%)			0	0	0	0	0	0	0	0	0
p-value			0.002*	0.000*	0.008*	0.000*	1.000	0.000*	1.000	1.000	0.000*

Antimicrobial agents: AML = Amoxicillin, AMP = Ampicillin, C = Chloramphenicol, CT = Colistin, CTX = Cefotaxime, DO = Doxycycline, FEP = Cefepime, FOX = Cefoxitin, SXT = Sulphamethoxazole + trimethoprim. Antimicrobial susceptibility: S = Susceptible, I = Intermediate susceptible, R = Resistant.

1 Cloacal swab,

2 Breast meat,

3 Resistant *Salmonella*,

4 Intestinal content.

* The prevalence of resistant *Salmonella* isolates between Thailand and Japan was significantly different (p < 0.05)

**Table-5:** MDR of *Salmonella* isolates.

Country	Sample	No. of *Salmonella* isolates	No. of AM^1^ classes/No. of AM						Total MDR

			3/3	3/4	4/4	4/5	4/6	5/6	
Thailand	CS^2^	96	8	3	8	10	1	3	33
	BM^3^	134	16	4	6	7	0	1	34
Japan	IC^4^	34	0	0	0	0	0	0	0
	BM	23	0	0	0	0	0	0	0

1 Antimicrobial,

2 Cloacal swab,

3 Breast meat,

4 Intestinal content. MDR=Multidrug resistance

**Table-6:** The prevalence of AMRGs in positive *Salmonella* isolates from chicken and chicken meat at local poultry slaughterhouses in Thailand and Japan.

Country	Sample	AMRGs (%)		

		ESBL-TEM^1^	*Int-1*	*mcr-1*
Thailand	CS^2^	10/23 (43.48)	3/23 (13.04)	2/23 (8.69)
	BM^3^	13/27 (46.43)	3/27 (10.71)	4/27 (14.28)
Japan	IC^4^	0/57 (0)	34/34 (100)	0/57 (0)
	BM	0/57 (0)	23/23 (100)	0/57 (0)

1 Extended-spectrum beta-lactamase–Temoneira,

2 Cloacal swab,

3 Breast meat,

4 Intestinal content. AMRGs=Antimicrobial resistance genes, *Int-1**=***Class 1 integron gene

## Discussion

In this study, the prevalence of *Salmonella* isolated from chicken and chicken meat from Thailand and Japan was 41.22% and 40.71%, respectively. Based on the small sample size from Japan, as well as time and regional study restrictions, the *Salmonella* prevalence was not comparable between Japan and Thailand. In this study, *Salmonella* prevalence during the pre-and post-slaughtering processes in Japan was similar to a previous study by Castro-Vargas *et al*. [16] that reported prevalence of 48.6% (vs. 40.5%) in broiler farms and 32.9% (vs. 30%) in broiler meat, respectively. Findings in this study suggest that *Salmonella* contamination may be caused by poor hygiene farm management practices rather than slaughtering processes. This concurs with previous studies by Sobur *et al*. [17] and Akter *et al*. [18], which reported that poor elimination of vectors (such as houseflies) in poultry farms led to increased *Salmonella* contamination. In addition, researchers have reported different *Salmonella* prevalences in Japan depending on region, climatic factors, and serovar findings [5]. This study revealed a higher prevalence of *Salmonella* serovar Schwarzengrund than Ishihara *et al*. [5], who found 23.3%, and Shimojima *et al*. [10] found 30.3%. These differences indicate that *S*. Schwarzengrund in chicken meat in Japan may have increased during the year between those studies and this study. In Thailand, the prevalence of *Salmonella* after slaughtering (breast meat samples) was higher than that before slaughtering (cloacal swab samples) (48.0% vs. 34.4%). This indicates that *Salmonella* contamination likely occurred during the slaughtering processes and may have come from the slaughterhouse environment and evisceration areas as indicated by the three most commonly identified serovars in this study of Corvallis, Enteritidis, and Typhimurium. These *Salmonella* serovars have been detected primarily in poultry farm environments and poultry meat [19, 20]. In addition, an effective biosecurity program used by broiler farms can decrease *Salmonella* cross-contamination among broilers, humans, the environment, and broiler meat during the slaughtering process [21].

In this study, most *Salmonella* isolates from Thailand showed phenotypic resistance to DO (84.4%) and CT (68.7%), while no *Salmonella* isolates from Japan had phenotypic resistance to the nine antibiotics tested. However, these strains exhibited phenotypically intermediate resistance (87.72% [50/57]) to DO, indicating that they may have increased resistance to the drug. These results demonstrated that tetracycline derivatives, such as DO or oxytetracycline, are being used and may be misused or overused for treating poultry diseases in farms in both countries. This finding is similar to those of previous studies by Duc *et al*. [6], Shimojima *et al*. [10] and Sornplang *et al*. [19]. In this study, the *Salmonella* strains from Thailand also had high phenotypic resistance to CT, which was the second-most common form of resistance. In Thailand, CT has been prohibited for preventing Gram-negative bacterial infections in farm animals since 2017, but it is allowed for short-term use in treating infections. In addition, Wongsuvan *et al*. [22] reported the routine use of CT as a feed additive in rural poultry farms in Thailand. Therefore, CT may be misused or overused on these farms.

This study tested two AMRGs (ESBL-TEM, *mcr-1*) and one mobile genetic unit *Int-1* commonly found in Gram-negative bacteria. *Mcr-1* is a colistin gene frequently found in pathogenic bacteria. The prevalence of genetic AMR in *Salmonella* isolates from Thailand in this study is consistent with phenotypic MDR, of which the most common was ESBL-TEM (45.10%), followed by the *Int-1 (*11.76%) and *mcr-1 (*11.76%) genes. This study showed a lower frequency of the ESBL gene than the previous study by Hosain *et al*. [23] (77.3%). This result may be due to sample size variations, geographic distribution, and *Salmonella* serovars.

The positive MDRS isolates from Thailand harbored all three genes, which remain concerning with respect to the antibiotics commonly used on poultry farms. In Japan, the positive *Salmonella* isolates only contained the *Int-1* gene. However, all the isolates were positive for *Int-1*. The *Int-1* is the most common genetic element that carries, captures, and shuffles antibiotic resistance genes embedded in gene cassettes and is widely found among Gram-negative bacteria [24]. Furthermore, it has been detected in *Salmonella* isolated from poultry associated with aminoglycoside resistance genes [25]. Our study found that chicken meat from a poultry slaughterhouse in Japan remained affected by antibiotic-resistant *Salmonella* isolates that may carry other AMRGs within the *Int-1*.

## Conclusion

This study recovered *Salmonella* spp. in 41.22% (230/558) of the Thai samples and 40.71% (57/140) of the Japanese samples, the latter of which were serovar Schwarzengrund in all cases. Most *Salmonella* isolates from Thailand were found to have phenotypic resistance to DO. Multidrug-resistant *Salmonella* analysis also identified AMRGs ESBL-TEM, *Int-1*, and *mcr-1*. In contrast, among the Japanese samples, the *Salmonella* isolates had no phenotypic resistance to any of the tested antibiotics. However, all these isolates contained the *Int-1* gene. Therefore, AMR *Salmonella* and MDRS isolates remain a public health concern, and stakeholders in both countries should increase monitoring and planning to allow for effective control of these resistant *Salmonella* isolates.

## Authors’ Contributions

PS and KD: Conceptualized and designed the study. PN: Sample collection, microbiological culture, and PCR. PS and PN: Drafted the manuscript and statistical analysis. PS and KD: Revised the manuscript. All authors have read, reviewed, and approved the final manuscript.
